# Mesenchymal stem cell conditioned medium attenuates oxidative stress injury in hepatocytes partly by regulating the miR-486-5p/PIM1 axis and the TGF-β/Smad pathway

**DOI:** 10.1080/21655979.2021.1972196

**Published:** 2021-09-14

**Authors:** Ning Ma, Shuo Li, Chao Lin, Xianbin Cheng, Zihui Meng

**Affiliations:** a Department of Hepatobiliary-Pancreatic Surgery, China-Japan Union Hospital of Jilin University, Changchun, Jilin, China; b Department of Gastrointestinal Colorectal and Anal Surgery, China-Japan Union Hospital of Jilin University, Changchun, Jilin, China

**Keywords:** Oxidative injury, UCB-MSC-CM, miR-486-5p, PIM1, TGF-β/Smad pathway

## Abstract

This study investigated the role of microRNA (miRNA) miR-486-5p in oxidative stress injury in hepatocytes under the treatment of mesenchymal stem cell conditioned medium (MSC-CM). The oxidative stress injury in hepatocytes (L02) was induced by H_2_O_2_. Human umbilical cord blood MSC-CM (UCB-MSC-CM) was prepared. The effects of UCB-MSC-CM on the proliferation, apoptosis, and inflammatory response in L02 cells were detected by Cell Counting Kit-8 (CCK-8) assay, flow cytometry analysis, and enzyme-linked immunosorbent assay (ELISA). Subsequently, the target of miR-486-5p was predicted using bioinformatics analysis, and the possible signaling pathway addressed by miR-486-5p was explored using western blot. We found that miR-486-5p expression was elevated following oxidative stress injury and was reduced after UCB-MSC-CM treatment. UCB-MSC-CM protected L02 cells against H_2_O_2_-induced injury by downregulation of miR-486-5p. Proviral integration site for Moloney murine leukemia virus 1 (PIM1) was verified to be targeted by miR-486-5p. UCB-MSC-CM upregulated the expression of PIM1 reduced by H_2_O_2_ in L02 cells. Additionally, silencing PIM1 attenuated the protective effects of miR-486-5p downregulation against oxidative stress injury. We further demonstrated that UCB-MSC-CM inhibited the TGF-β/Smad signaling in H_2_O_2-_treated L02 cells by the miR-486-5p/PIM1 axis. Overall, UCB-MSC-CM attenuates oxidative stress injury in hepatocytes by downregulating miR-486-5p and upregulating PIM1, which may be related to the inhibition of TGF-β/Smad pathway.

## Introduction

Oxidative stress injury is a major risk factor that leads to severe local and remote tissue injury and subsequent distant organ functional failure, including that of spinal cord, kidney, cardiac vessel, and liver [1–4]. Oxidative stress is a common pathophysiological basis of diverse liver diseases [5,6], and displays important roles in fatty liver, viral hepatitis, and liver fibrosis [7–9]. To date, no effective therapeutic strategies have been proven to modify the course of oxidative stress injury.

Increasing evidence has revealed the significant potentials of mesenchymal stem cells (MSCs) in repairing ischemic organ injury [10]. Studies have demonstrated that MSC conditioned medium (MSC-CM) is a promising therapeutic agent to promote cell proliferation and inhibit cell apoptosis and inflammation after acute organ damage [11–13]. CM is heterogeneous and contains various soluble factors. UCB-MSC-CM injection can stimulate hepatocyte regeneration and reduce hepatocyte apoptosis in animals with liver failure [14–16]. More importantly, a previous study demonstrated a favorable tendency toward survival caused by MSC‐CM treatment [17]. Here, we explored the function of UCB-MSC-CM in oxidative stress-induced injury.

MicroRNAs (miRNAs), a group of short noncoding nucleotides that control mRNA translation at the posttranscriptional level, play critical roles in mediating physiological processes, including cell growth, apoptosis, and differentiation [18–20]. The regulatory effects of mature miRNAs are shown in numerous pathological processes [21]. UCB-MSC-CM has the potential of controlling miRNA expression, thus inducing alteration in cellular microenvironments [20]. Previous research showed the important functions of miR-486-5p in regulating hepatocellular carcinoma development. MiR-486-5p prevents cell viability, migration, and invasion in hepatocellular carcinoma by binding to phosphoinositide 3-kinase regulatory subunit 1 [22]. MiR-486-5p attenuates the malignancy of hepatocellular carcinoma by suppressing Casitas B-lineage lymphoma [23]. In addition, downregulation of miR-486-5p alleviates acute lung injury by inhibiting apoptosis through upregulating OTU-domain-containing 7B [24]. However, the biological role of miR-486-5p in hepatocyte injury is unclear.

Until now, little is known about the expression change of miRNAs in hepatocytes during the MSC-CM-mediated process. Here, miR-486-5p was shown to be downregulated after UCB-MSC-CM treatment. We hypothesized that UCB-MSC-CM could attenuate oxidative stress injury in hepatocytes by regulating miR-486-5p expression. This study was aimed to explore the role of miR-486-5p in cell proliferation, apoptosis, and inflammation in H_2_O_2_-treated hepatocytes in the presence of UCB-MSC-CM as well as the possible mechanisms involved, which may provide some theoretical insight into further exploration of molecular mechanisms related to the therapeutic effects of MSC-CM on hepatocyte injury.

## Materials and methods

### Culture of UCB-MSCs

Umbilical cord blood-derived MSCs (UCB-MSCs) were obtained from the umbilical cord of a pregnant woman who underwent a cesarean section and signed informed consent at China-Japan Union Hospital of Jilin University. This study was approved by the Ethics Committee of China-Japan Union Hospital of Jilin University (approval number: 2,021,081,012). Mononuclear cells, collected by Ficoll-Paque density gradient centrifugation (GE Healthcare, Pittsburgh, PA, USA), were incubated in Dulbecco’s modified Eagle medium (Gibco; Thermo Fisher Scientific, MA, USA) containing antibiotics (100 U/mL of penicillin and 100 U/mL of streptomycin) and 10% fetal bovine serum (Gibco) in a humidified incubator at 37°C with 5% CO_2_. The medium was refreshed every 2–3 days. The UCB-MSCs between passage 3 and 6 were used in this study.

### Preparation of UCB-MSC-CM

When the culture reached 70–80% confluency, UCB-MSCs were washed three times with phosphate buffered saline and the medium was replaced with a fresh complete medium. Subsequently, UCB-MSC-CM was centrifuged at 2500 rpm for 20 minutes to remove any cell debris and passed through a 0.22 μm of filter. The resulting UCB-MSC-CM was then diluted with fresh high-glucose Dulbecco’s modified Eagle medium to achieve a final concentration of 50% [25].

### Cell surface antigen phenotyping

The characteristics of cultured UCB-MSCs at passage 3 were identified by flow cytometry. As previously published [26], cells were treated with 1 mL of trypsin-Ethylene Diamine Tetraacetic Acid at 37°C for 5–10 minutes. Cells were then resuspended in 10 mL of phosphate buffered saline and centrifuged at 1,000 × g for 5 minutes. After that, cells were resuspended in flow cytometry buffer (phosphate buffered saline containing 2 mM of Ethylene Diamine Tetraacetic Acid and 10% blocking reagent) at 1 × 10^6^ cells/mL. Next, 50–100 µL of cell suspension was added to a 1.5 mL of tube and incubated with 2 µL of fluorescent antibodies (CD29, CD34, CD45, CD105; BD Pharmingen, United States) and the homotypic controls on ice for 45 minutes. Next, cells were washed with flow cytometry buffer, fixed in 10% formalin, and stained with 50–100 µL of 0.2% viability dye solution. After incubation at room temperature for 15 minutes, cells were washed with flow cytometry buffer twice and filtered through a 70 µm of cell strainer. The positive rate of antigen was analyzed by a flow cytometry system (Guava easyCyte8HT, EMD Millipore, Billerica, MA).

### Adipogenic and osteogenic differentiation

UCB-MSCs were seeded into 6-well plates (Corning Inc., Corning, NY) at 2  ×  10^4^ cells/well, in adipogenic induction medium containing 10% fetal bovine serum (Gibco), 1 μM of dexamethasone, 100 μM of indomethacin, 500 μM of 3-isobutyl-1-methylxanthine, and 10 μg/mL of insulin (all from Sigma-Aldrich, St Louis, MO). The fresh medium was replaced every 3 days. After 21 days, Oil Red-O (Sigma-Aldrich) staining was used to identify intracellular accumulation of lipid-rich vacuoles. Briefly, cells were fixed with 4% paraformaldehyde for 30 minutes, washed with phosphate buffered saline, and stained with a working solution of 0.3% Oil Red-O in phosphate buffered saline for 20  minutes. For osteogenesis, after preparation with 0.1 µM of dexamethasone (Sigma-Aldrich), 0.2 µM of ascorbic acid 2-phosphate (Sigma-Aldrich), 10 mM of glycerol 2-phosphate (Sigma-Aldrich), and 10% fetal bovine serum, and fixation cells were stained with Alizarin red S (Fluka Buchs SG, Switzerland) [27].

### Hepatic cell culture and processing

Human normal liver cell line L02 (the American Type Culture Collection; MD, USA) was maintained in Dulbecco’s modified Eagle medium containing antibiotics (100 U/mL of penicillin and 100 U/mL of streptomycin) and 10% fetal bovine serum (Gibco) in a humidified incubator at 37°C with 5% CO_2_. When the culture reached 80%, 1 mM of H_2_O_2_ (Sigma-Aldrich) was used to treat cells for 4 h to induce oxidative stress injury. Subsequently, cells were divided into 3 groups: the control group, the H_2_O_2_ group (treatment with 1 mM of H_2_O_2_ for 4 h), and the H_2_O_2_ + UCB-MSC-CM group (treatment with 30% UCB-MSC-CM for 6, 24, and 48 h after H_2_O_2_ stimulation). Cell Counting Kit-8 (CCK-8) assay was used to detect the optimal time of UCB-MSC-CM, which was determined to be 24 h.

### Cell transfection and grouping

The oligonucleotides including miR-486-5p mimics (UCCUGUACUGAGCUGCCCCGAG), miR-486-5p inhibitor (CUCGGGGCAGCUCAGUACAGGA), their negative controls NC mimics (UAUCCGGCCUGCGCCGUUAGCA), NC inhibitor (ACCUAUCUGCGAAGGCCGGAGG), as well as the small interference RNA (siRNA) targeting Proviral integration site for Moloney murine leukemia virus 1 (PIM1) (si-PIM1, GACUUAGGAUGUUGUGCAAGC), and si-NC (AAGUGAGCGUUAGGCCUUGUA) were designed and synthesized by GenePharma (Shanghai, China). Transfection was performed using Lipofectamine 3000 (Invitrogen, CA, USA) following the manufacturer’s instructions as described previously [28]. Briefly, L02 cells during logarithmic growth were treated with 0.25% trypsin and seeded in 6-well plates at 1 × 10^5^ cells/well. Next, miR-486-5p mimics (50 nM), miR-486-5p inhibitor (50 nM), and negative controls (at a final concentration of 50 nM) were transfected into cells using Lipofectamine 3000 (Invitrogen) following the manufacturer’s instructions. Cells were incubated at 37°C for 48 h and then collected. Afterward, L02 cells were treated with 0.8 mM of H_2_O_2_ for 3 h for detection of cell proliferation, apoptosis, and inflammatory response.

In detail, the transfected L02 cells were divided into 7 groups: the control group, the H_2_O_2_ group (treatment with 0.8 mM of H_2_O_2_ for 3 h), the H_2_O_2_ + NC group (treatment with 0.8 mM of H_2_O_2_ for 3 h + NC), the H_2_O_2_ + miR-486-5p mimics group (treatment with 0.8 mM of H_2_O_2_ for 3 h + miR-486-5p mimics), the H_2_O_2_ + miR-486-5p inhibitor group (treatment with 0.8 mM of H_2_O_2_ for 3 h + miR-486-5p inhibitor), the H_2_O_2_ + si-PIM1 group (treatment with 0.8 mM of H_2_O_2_ for 3 h + si-PIM1) and the H_2_O_2_ + miR-486-5p inhibitor + si-PIM1 group (treatment with 0.8 mM of H_2_O_2_ for 3 h + miR-486-5p inhibitor + si-PIM1).

### Cell Counting Kit-8 (CCK-8)

As previously described [29], the transfected L02 cells were seeded in 96-well plates at 5 × 10^3^ cells/well. CCK-8 reagent (10 µL; Dojin Laboratories, Japan) was added to each well at 24 h, and then incubated for another 4 h at 37°C. Cell viability was detected by measuring the optical density (OD) value at 450 nm using a Microplate Reader (Bio-Rad, USA).

### Flow cytometry

Annexin V-fluoresceine isothiocyanate (FITC)/Prodium Iodide (PI) double-labeled staining kit (Sigma-Aldrich) was used to detect apoptosis. The procedure was performed as previously described [30]. The transfected L02 cells were treated with 0.25% Ethylene Diamine Tetraacetic Acid-free trypsin, followed by centrifugation at 2000 rpm for 15 minutes. Cells were then resuspended in pre-cooled phosphate buffered saline and centrifuged again at 2000 rpm for 15 minutes. After washing, cells were resuspended in 300 μL of binding buffer, and then stained with 10 μL of Annexin V-FITC and 5 μL of PI at room temperature for 10 minutes in the dark. Finally, cell apoptosis was assessed using a BD FACS Calibur Flow Cytometer (Beckman Coulter, USA). Data analysis was performed using Guava Incyte (EMD Millipore, USA).

### Cytokine measurement

Enzyme-linked immunosorbent assay (ELISA) was carried out to detect the levels of tumor necrosis factor alpha (TNF-α), interleukin-6 (IL-6), and interleukin-1β (IL-1β) in L02 cells utilizing commercial ELISA kits (Sigma-Aldrich) according to the manufacturer’s instructions.

### Reverse transcription quantitative polymerase chain reaction (RT-qPCR)

TRIzol reagent (Invitrogen) was used to extract total RNA from L02 cells. RNA was reverse transcribed into cDNA using a High Capacity cDNA Reverse Transcription Kit (Applied Biosystems Inc., Foster City, CA, USA). Real-time PCR was performed on ABI 7500-fast Real Time PCR system (Applied Biosystems Inc.) with PowerUp SYBR Green Master mix (Applied Biosystems Inc.) in 20 µL of PCR reaction. The thermocycling conditions of the qPCR reaction were initial denaturation at 95°C for 5 minutes, followed by 40 cycles of 95°C for 30 seconds and 60°C for 45 seconds. For RT-qPCR analysis, GAPDH and U6 were used as internal controls. The expression was analyzed by the 2^−ΔΔCt^ method [31]. The used primers are as follows:

miR-486-5p: Forward, 5ʹ-CTCGCTTCGGCAGCACA-3ʹ

miR-486-5p: Reverse, 5ʹ-ACGCTTCACGAATTTGCGT-3ʹ

PIM1: Forward, 5ʹ-TCATTAGATGGTGCTTGGCCCTGA-3ʹ

PIM1: Reverse, 5ʹ-TGTGGAGGTGGATCTCAGCAGCAGTTT-3ʹ

GAPDH: Forward, 5ʹ-ACTGAGCAAGAGAGGCCCTA-3ʹ

GAPDH: Reverse, 5ʹ- TATGGGGGTCTGGGATGGAA-3ʹr

U6: Forward, 5ʹ-CTCGCTTCGGCAGCACA-3ʹ

U6: Reverse, 5ʹ-AACGCTTCACGAATTTGCGT-3ʹ

### Western blot analysis

Western blot was performed using standard and established protocol as previously published [32]. Proteins were extracted from L02 cells using radioimmunoprecipitation assay lysis buffer (Beyotime, Shanghai, China). Protein concentration was quantified using a bicinchoninic acid assay kit (Beyotime), and an equal amount of proteins were loaded and run on 12% sodium dodecyl sulfate-polyacrylamide gel electrophoresis. Proteins were then transferred onto polyvinylidene difluoride membranes (Millipore, Billerica, MA, USA). After being blocked with 0.5% defatted milk, proteins were examined using western blot with the specific antibodies. The primary antibodies include anti-cleaved caspase3 (ab2302; 1:1000), anti-cleaved caspase-9 (ab2324; 1:1000), anti-TGF-β (ab92486; 1:1000), anti-p-Smad3 (ab52903; 1:1000), anti-Smad3 (ab227223; 1:1000), and anti-β-actin (ab8227; 1:5000). Then, the membranes were incubated with the corresponding secondary antibodies for 2 h and developed using Abnova Enhanced Chemiluminescence detection kits (Millipore). The images were analyzed using ImageJ software.

### Luciferase reporter assay

The wild type (Wt) or mutant (Mut) sequence in the PIM1 3ʹUTR containing the predicted binding site for miR-486-5p was synthesized to generate the fragment of PIM1-wild type (PIM1-Wt) and fragment of PIM1-mutant (PIM1-Mut). The targeted fragments were inserted into the pmirGLO vector (Promega, Madison, WI, USA). Then the recombinant plasmids (PIM1-Wt and PIM1-Mut) were transfected with miR-486-5p mimics or NC mimics into 293 T cells, respectively. After 48 h, the luciferase activity was examined using the Dual-luciferase Reporter Assay System (Promega) [29].

### Statistical analysis

The experimental data are shown as the mean ± standard deviation. Statistical analysis was performed using SPSS20.0 statistics program (IBM, USA). For data conforming to normal distribution and homogeneity of variance, the paired t-test was employed to compare data within a group, while the unpaired t-test was used for comparisons between two groups. One-way analysis of variance followed by Tukey’s post hoc test was adopted for comparison among multiple groups. *P* < 0.05 was statistically significant.

## Results

We hypothesized that UCB-MSC-CM could attenuate oxidative stress injury in hepatocytes by regulating miR-486-5p expression. This study was aimed to explore the role of miR-486-5p in cell proliferation, apoptosis, and inflammation in H_2_O_2_-treated hepatocytes in the presence of UCB-MSC-CM as well as the possible mechanisms involved. We examined the proliferation, apoptosis, and inflammatory response in L02 cells. Our results showed that miR-486-5p expression was elevated in L02 cells following oxidative stress injury and was reduced after UCB-MSC-CM treatment. UCB-MSC-CM attenuates oxidative stress injury in hepatocytes by inhibiting miR-486-5p and upregulating PIM1, which may be related to the inhibition of TGF-β/Smad pathway.

### Characteristics and differentiation of UCB-MSCs

**Figure 1.**
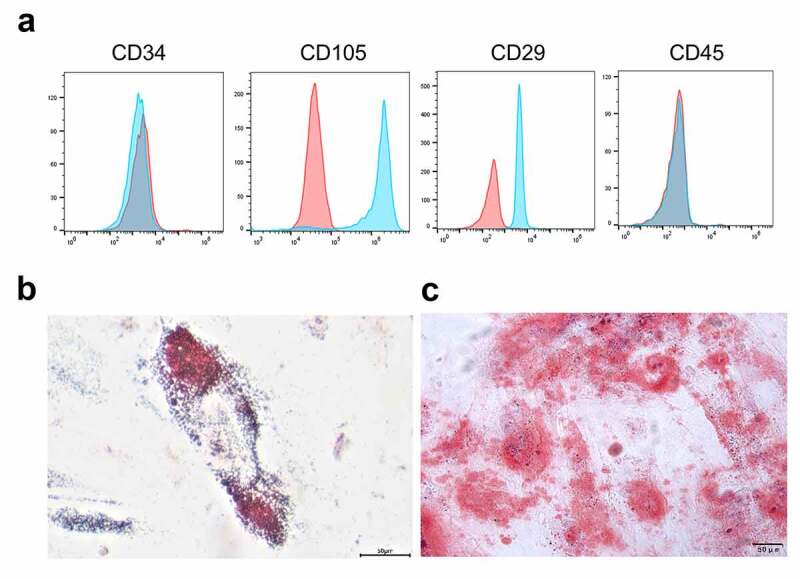
Characteristics and differentiation of UCB-MSCs. (a) Immunophenotyping of UCB-MSCs. Analysis of MSC markers including CD105 and CD29, as well as hematopoietic markers CD34 and CD45 was performed by flow cytometry. (b) Adipogenic differentiation detected by Oil Red O staining. (c) Osteogenic differentiation detected by Alizarin Red staining. Scale bars: 50 µm. UCB-MSCs: umbilical cord blood mesenchymal stem cells

As indicated by flow cytometry analysis in Figure 1(a), antigen profiling of the UCB-derived MSCs showed the high expression of positive stromal markers (CD105 and CD29), as well as the absence of negative hematopoietic markers (CD34 and CD45), suggesting that UCB-MSCs share common immunophenotypes with MSCs. Furthermore, the characteristics of UCB-MSCs were confirmed by osteogenic and adipogenic differentiation assays. For adipogenic differentiation, the intracellular lipid droplets stained by Oil Red O could be observed (Figure 1(b)). Osteogenic differentiation caused calcium deposits, as shown by Alizarin Red staining (Figure 1(c)). These findings indicated that UBC-derived MSCs are characterized by the capacities of stem cells.

### UCB-MSC-CM protects L02 cells against H_2_O_2_-induced injury by suppressing miR-486-5p

**Figure 2.**
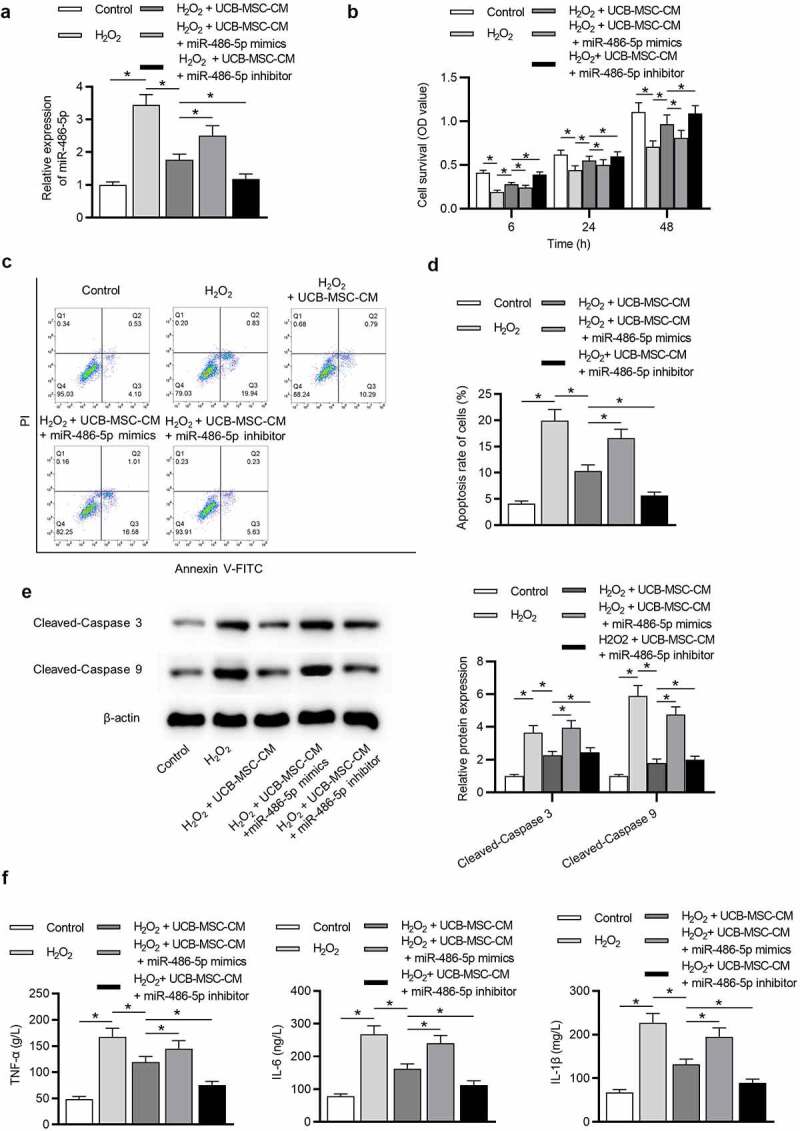
UCB-MSC-CM protects L02 cells against oxidative injury by miR-486-5p. (a) RT-qPCR was used to measure the expression of miR-486-5p in L02 cells after H_2_O_2_ stimulation and UCB-MSC-CM treatment. (b) CCK-8 assay was performed to detect the effects of UCB-MSC-CM after overexpression or downregulation of miR-486-5p on L02 cell proliferation. (c-d) Flow cytometry analysis revealed the effects of UCB-MSC-CM after overexpression or downregulation of miR-486-5p on L02 cell apoptosis. (e) Western blot analysis was performed to evaluate the influences of UCB-MSC-CM after overexpression or downregulation of miR-486-5p on proapoptotic proteins. (f) ELISA was conducted to measure the levels of inflammatory cytokines in each group. *P < 0.05

RT-qPCR indicated that miR-486-5p expression was elevated in the H_2_O_2_ group while downregulated in the UCB-MSC-CM group (Figure 2(a), p < 0.05). We further overexpressed and downregulated miR-486-5p expression in H_2_O_2_-treated L02 cells using miR-486-5p mimics and miR-486-5p inhibitor, respectively (Figure 2(a), p < 0.05). Subsequent assays were performed to determine the effects of UCB-MSC-CM after miR-486-5p overexpression or downregulation in L02 cells treated with H_2_O_2_. As shown by CCK-8 assays in Figure 2(b), H_2_O_2_ stimulation significantly inhibited the proliferation, while the proliferation was restored after UCB-MSC-CM treatment. Overexpression of miR-486-5p eliminated the promotive effects of UCB-MSC-CM on cell proliferation, while downregulation of miR-486-5p further promoted L02 cell proliferation. Data from flow cytometry revealed that UCB-MSC-CM effectively inhibited L02 cell apoptosis stimulated by H_2_O_2_. MiR-486-5p overexpression restored cell apoptosis, while miR-486-5p downregulation further suppressed apoptosis of L02 cells (Figure 2(c-d), p < 0.05). Western blot analysis showed that UCB-MSC-CM reduced the levels of Cleaved-Caspase 3/9 upregulated by H_2_O_2_, and miR-486-5p inhibition further reduced the levels of these proteins. MiR-486-5p upregulation had an opposite effect (Figure 2(e), p < 0.05). The results from ELISA demonstrated that UCB-MSC-CM decreased the levels of TNF-α, IL-6, and IL-1β in the H_2_O_2_ group. Overexpression of miR-486-5p restored the levels of these cytokines, and downregulation of miR-486-5p further suppressed the levels of these cytokines (figure 2(f), p < 0.05).

### Silencing miR-486-5p ameliorates H_2_O_2_-induced L02 cell injury

**Figure 3.**
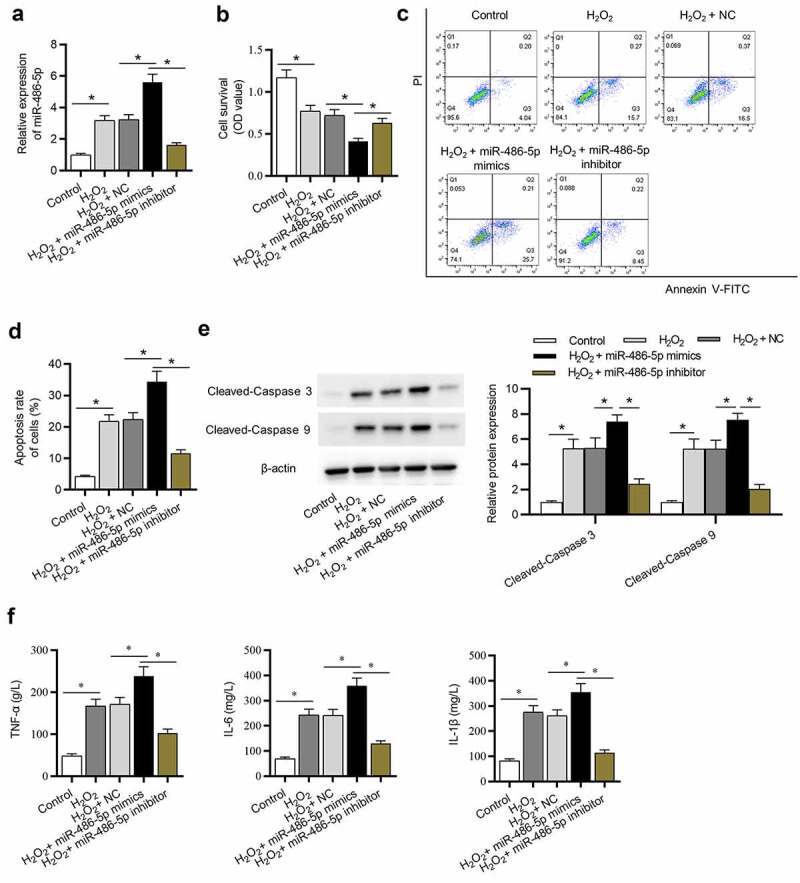
Downregulation of miR-486-5p ameliorates H_2_O_2_-induced L02 cell injury. (a) The expression of miR-486-5p in each group was determined by RT-qPCR. (b) Cell proliferation in each group was assessed by CCK-8. (c-d) Cell apoptosis in each group was detected by flow cytometry analysis. (e) The levels of proapoptotic proteins were measured using western blot analysis. (f) The levels of inflammatory cytokines were detected using ELISA. *P < 0.05

As displayed in Figure 3(a) (p < 0.05), no significant difference of miR-486-5p levels was detected between the H_2_O_2_ group and H_2_O_2_ + NC group. MiR-486-5p expression was elevated in L02 cells with miR-486-5p mimics and decreased with miR-486-5p inhibitor. CCK-8 assay exhibited that miR-486-5p mimics impeded the proliferation of H_2_O_2_-treated L02 cells while miR-486-5p inhibitor exerted an opposite effect (Figure 3(b), p < 0.05). The apoptosis was increased after miR-486-5p overexpression but decreased after miR-486-5p downregulation (Figure 3(c-d), p < 0.05). Furthermore, we found that the levels of proapoptotic proteins were increased in the H_2_O_2_ group + miR-486-5p mimics group and decreased in the H_2_O_2_ group + miR-486-5p inhibitor group (Figure 3(e), p < 0.05). Additionally, miR-486-5p mimics elevated the levels of proinflammatory cytokines while miR-486-5p inhibitor exerted an opposite effect (figure 3(f), p < 0.05).

### PIM1 is targeted by miR-486-5p

**Figure 4.**
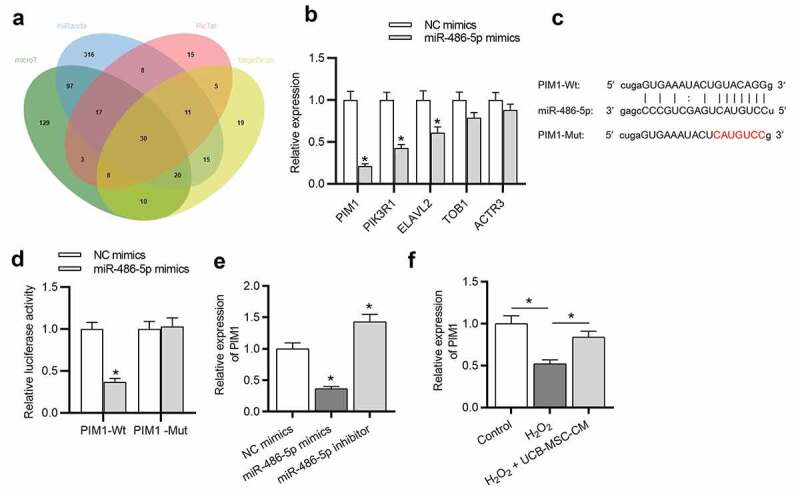
PIM1 is targeted by miR-486-5p. (a) Bioinformatics tools (microT, miRanda, PicTar and TargetScan) were used and Venn diagram exhibits 30 potential targets of miR-486-5p. (b) After miR-486-5p overexpression, the expression of targets was measured by RT-qPCR. (c) MiR-486-5p binding site in the wild type or mutant sequence of PIM1-3ʹUTR. (d) The binding capacity between miR-486-5p and PIM1 was confirmed by a luciferase reporter assay. (e) The expression of PIM1 in H_2_O_2_-treated cells transfected with miR-486-5p mimics or inhibitor was measured by RT-qPCR. (f) RT-qPCR was used to measure the expression of PIM1 in L02 cells after H_2_O_2_ stimulation and UCB-MSC-CM treatment. *P < 0.05

To identify the downstream targets of miR-486-5p, bioinformatics tools (microT, miRanda, PicTar and TargetScan) were used. Venn diagram shows 30 candidates that have predicted binding site for miR-486-5p (Figure 4(a)). We found that PIM1 expression showed the most significant downregulation after miR-486-5p overexpression (Figure 4(b), p < 0.05). Thus, PIM1 was chosen for the further study. The binding site between miR-486-5p and PIM1 is displayed in Figure 4(c). Subsequently, luciferase reporter assay was carried out to verify the binding between PIM1 and miR-486-5p. MiR-486-5p overexpression suppressed the luciferase activity of PIM1-Wt reporters, while that of PIM1-Mut reporters showed no response to miR-486-5p overexpression (Figure 4(d), p < 0.05). The expression of PIM1 in L02 cells transfected with miR-486-5p mimics or inhibitor was determined using RT-qPCR. As Figure 4(e) (p < 0.05) shows, PIM1 expression was decreased by miR-486-5p mimics and increased by miR-486-5p inhibitor, suggesting that miR-486-5p negatively regulates PIM1 expression. Additionally, UCB-MSC-CM treatment restored the PIM1 expression reduced by H_2_O_2_ (figure 4(f), p < 0.05).

### MiR-486-5p ameliorates H_2_O_2_-induced L02 cell injury by upregulating PIM1

**Figure 5.**
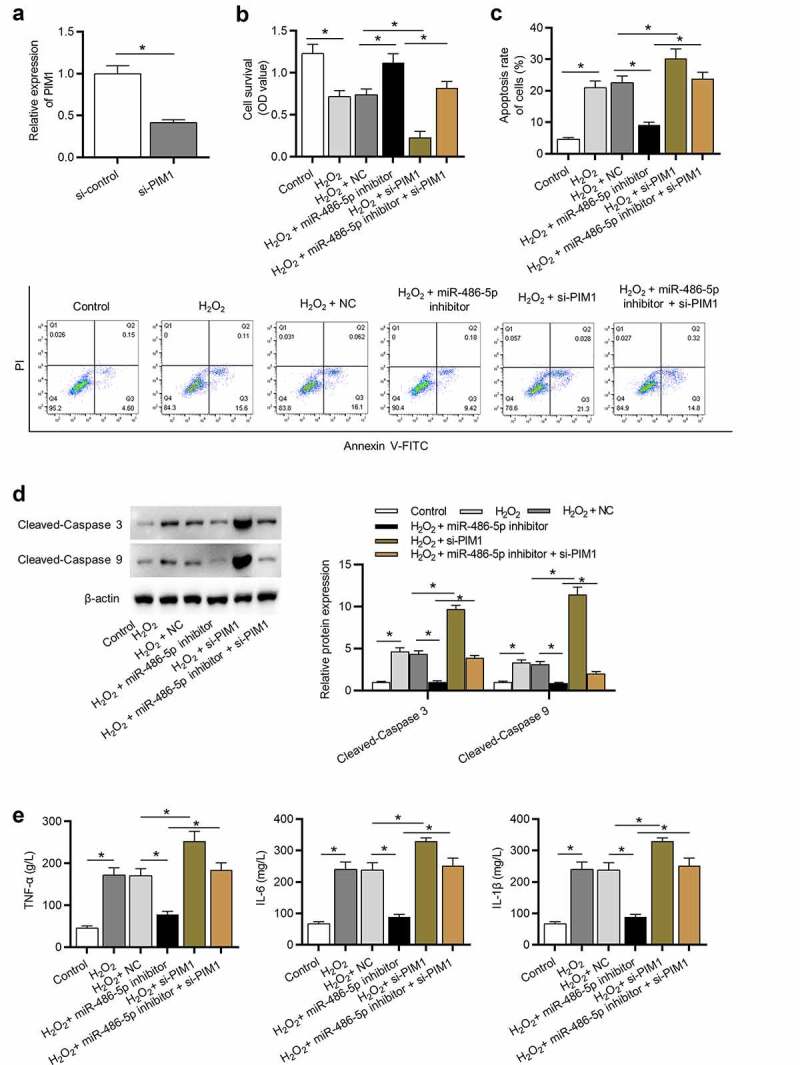
MiR-486-5p ameliorates H_2_O_2_-induced injury in L02 cells by upregulating PIM1. (a) PIM1 expression in cells transfected with si-control or si-PIM1 was analyzed by RT-qPCR. (b) Cell proliferation in each group was determined using CCK-8. (c) Cell apoptosis in each group was assessed by flow cytometry. (d) The levels of pro-apoptotic proteins were measured using western blot analysis. (e) The levels of inflammatory cytokines were detected by ELISA. *P < 0.05

We then investigated whether miR-486-5p affects L02 cell injury by regulation of PIM1. PIM1 expression in L02 cells was effectively knocked down after transfection of si-PIM1 (Figure 5(a), p < 0.05). The results of functional assays showed that PIM1 knockdown antagonized the effects mediated by miR-486-5p downregulation on cell proliferation, apoptosis, and inflammatory response H_2_O_2_-treated cells, as demonstrated by a significant reduction in cell proliferation ability (Figure 5(b), p < 0.05), a significant increase in cell apoptosis (Figure 5(c-d); p < 0.05), and a significant increase in concentrations of pro-inflammatory cytokines (Figure 5(e), p < 0.05).

### Downregulation of miR-486-5p inactivates TGF-β/Smad signaling by regulation of PIM1

**Figure 6.**
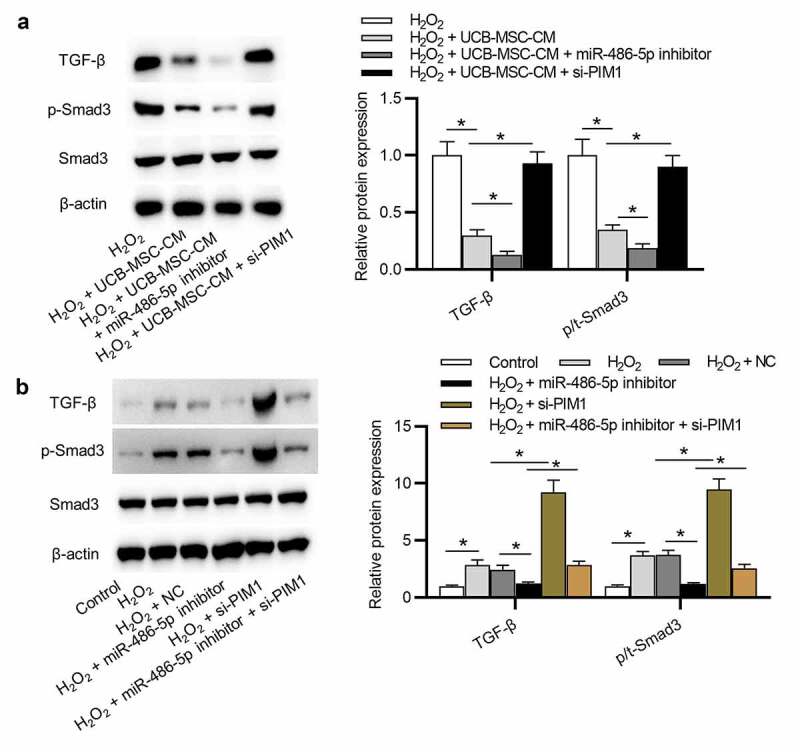
Downregulation of miR-486-5p inactivates the TGF-β/Smad pathway by regulation of PIM1. (a) The levels of TGF-β and phosphorylated (and total) Smad3 in L02 cells in 4 groups: H_2_O_2_; H_2_O_2_ + UCB-MSC-CM; H_2_O_2_ + UCB-MSC-CM + miR-486-5p inhibitor; H_2_O_2_ + UCB-MSC-CM + si-PIM1 were detected using western blot analysis. (b) The levels of TGF-β and phosphorylated (and total) Smad3 in L02 cells in 6 groups: Control; H_2_O_2_; H_2_O_2_ + NC; H_2_O_2_ + miR-486-5p inhibitor; H_2_O_2_ + si-PIM1; H_2_O_2_ + miR-486-5p inhibitor+si-PIM1 groups. *P < 0.05

A previous study indicated that PIM1 delays cellular senescence in cardiomyocytes by inhibiting TGF-β/Smad signaling [33]. We thus examined the expression levels of key signaling proteins of TGF-β/Smad signaling. As presented in Figure 6(a) (p < 0.05), the protein levels of TGF-β and phosphorylated Smad3 (p-Smad3) were elevated in the H_2_O_2_ group, showing that TGF-β/Smad signaling is activated after H_2_O_2_ stimulation. However, UCB-MSC-CM blocked TGF-β/Smad signaling; miR-486-5p inhibitor further inhibited TGF-β/Smad signaling. Additionally, the impact of miR-486-5p inhibitor in TGF-β/Smad signaling was reversed by PIM1 knockdown. These data demonstrated that UCB-MSC-CM inhibits TGF-β/Smad signaling by the miR-486-5p/PIM1 axis. Furthermore, the reverse effects of PIM1 knockdown on miR-486-5p were also reflected without UCB-MSC-CM (Figure 6(b), p < 0.05).

## Discussion

Increasing studies have revealed the close relationship between abnormal expression of miRNAs and hepatocyte injury [34,35]. UCB-MSC-CM plays a crucial role in repairing damaged cells by regulating the expression of miRNAs and changing the cell microenvironment [20]. In this work, the protective effects of UCB-MSC-CM against oxidative stress injury in hepatocytes was verified. Our study showed that UCB-MSC-CM protected hepatocytes against oxidative injury by mediating proliferation, apoptosis, inflammation in a miR-486-5p-dependent manner. Evidence demonstrates the significant roles of miRNAs regulated by UCB-MSC-CM in injury in myocardium [36], brain [37], and kidney [38]. A previous study indicates that silencing miR-486-5p alleviates acute lung injury by inhibiting apoptosis and inflammation via targeting OTU domain-containing protein 7B [24]. MiR-486-5p derived from exosomes of MSCs suppresses cardiomyocyte apoptosis under hypoxic impairment by activating the PTEN/PI3K/AKT pathway [39]. The present study revealed that downregulation of miR-486-5p promoted hepatocyte proliferation and inhibited H_2_O_2_-induced apoptosis and inflammation, suggesting the protective role of silencing miR-486-5p against oxidative stress injury in hepatocytes.

In the present study, PIM1 was predicted as a target of miR-486-5p using bioinformatics analysis. PIM1 has been found to participate in the progression of a variety of diseases. For example, PIM1 acts as an oncogenic gene in lung adenocarcinoma and promotes tumor growth by activating the c-mesenchymal to epithelial transition factor signaling pathway [40]. PIM1 promotes cell invasion, epithelial to mesenchymal transition process, and cancer cell stemness in IL-6-treated breast cancer cells [41]. PIM1 prevents oxidative stress and apoptosis in cardiomyocytes after exposure to hypoxia by promoting cell autophagy [42]. Additionally, PIM1 inhibits cellular senescence in cardiomyocytes by inhibiting TGF-β/Smad pathway [33]. In this study, PIM1 was identified to be a functional target of miR-486-5p and was downregulated in H_2_O_2_-treated hepatocytes. UCB-MSC-CM attenuated the damaging effects of H_2_O_2_ by upregulating PIM1 expression. Moreover, PIM1 knockdown reversed the protective effects of miR-486-5p downregulation on oxidative stress injury in H_2_O_2_-treated hepatocytes.

The transforming growth factor-β/Smad (TGF-β/Smad) is an important intracellular pathway that regulates multiple pathological processes such as cell growth, apoptosis, fibrosis, inflammation, and differentiation [43–46]. Accumulating research has demonstrated that the TGF-β/Smad signaling participates in various inflammatory diseases. Genistein ameliorates chronic liver injury induced by D-galactosamine in rats through suppressing TGF-β/Smad pathway [47]. Tanshinone IIA alleviates renal fibrosis and inflammation by blocking TGF-β/Smad pathway [48]. Fangxiao Formula inhibits TGF-β/Smad3 signaling pathway to alleviate airway inflammation and remodeling in rats with asthma [49]. In the present study, we found that the TGF-β/Smad3 pathway was stimulated by H₂O₂, while UCB-MSC-CM inactivated TGF-β/Smad3 pathway by downregulation of miR-486-5p. PIM1 knockdown reversed the effects of miR-486-5p downregulation on UCB-MSC-CM mediated-TGF-β/Smad3 signaling pathway. These results suggested that TGF-β/Smad3 pathway may be involved in the UCB-MSC-CM-mediated processes in L02 cells.

## Conclusion

In summary, this study shows that UCB-MSC-CM has significant potential to alleviate H_2_O_2_-induced oxidative stress injury and demonstrates for the first time that miR-486-5p is involved in the molecular mechanisms underlying the therapeutic effects of UCB-MSC-CM on oxidative stress injury in hepatocytes by controlling PIM1 and TGF-β/Smad3 signaling. There are still limitations in this study. Future study endeavors are required to investigate the precise regulatory mechanisms of how miR-486-5p exert its functions in the MSC-CM-mediated protection of hepatocytes against oxidative stress. More importantly, in vivo experiments are needed to validate the results of in vitro studies.

## Data Availability

The datasets used during the current study are available from the corresponding author on reasonable request.

## References

[1] Liu SG, Wang YM, Zhang YJ, et al. ZL006 protects spinal cord neurons against ischemia-induced oxidative stress through AMPK-PGC-1alpha-Sirt3 pathway. Neurochem Int. 2017;108:230–237. 28411102 10.1016/j.neuint.2017.04.005

[2] Amini N, Sarkaki A, Dianat M, et al. Protective effects of naringin and trimetazidine on remote effect of acute renal injury on oxidative stress and myocardial injury through Nrf-2 regulation. Pharmacol Rep. 2019 Nov;71(6):1059–1066. 31604166 10.1016/j.pharep.2019.06.007

[3] Lorenzon Dos Santos J, Quadros AS, Weschenfelder C, et al. Oxidative stress biomarkers, nut-related antioxidants, and cardiovascular disease. Nutrients. 2020 Mar 3;12(3):682. 10.3390/nu12030682 PMC7146201 32138220

[4] Wu W, Wang T, Sun B, et al. Xian-Ling-Gu-Bao induced inflammatory stress rat liver injury: inflammatory and oxidative stress playing important roles. J Ethnopharmacol. 2019 Jul;15(239):111910. 10.1016/j.jep.2019.111910 31026554

[5] Medina J, Moreno-Otero R. Pathophysiological basis for antioxidant therapy in chronic liver disease. Drugs. 2005;65(17):2445–2461. 16296871 10.2165/00003495-200565170-00003

[6] Wu Y, Zhao M, Lin Z. Pyrroloquinoline quinone (PQQ) alleviated sepsis-induced acute liver injury, inflammation, oxidative stress and cell apoptosis by downregulating CUL3 expression. Bioengineered. 2021;Dec;12(1):2459–2468. 10.1080/21655979.2021.1935136 PMC8806920 34227919

[7] Yang J, Fernandez-Galilea M, Martinez-Fernandez L, et al. Oxidative stress and non-alcoholic fatty liver disease: effects of omega-3 fatty acid supplementation. Nutrients. 2019 Apr 18; 11(4):872. 10.3390/nu11040872 PMC6521137 31003450

[8] Shahid M, Idrees M, Butt AM, et al. Blood-based gene expression profile of oxidative stress and antioxidant genes for identifying surrogate markers of liver tissue injury in chronic hepatitis C patients. Arch Virol. 2020 Feb 27;165(4):809-822. 10.1007/s00705-020-04564-z 32103340

[9] Shi H, Shi A, Dong L, et al. Chlorogenic acid protects against liver fibrosis in vivo and in vitro through inhibition of oxidative stress. Clin Nutr. 2016 Dec;35(6):1366–1373. 27017478 10.1016/j.clnu.2016.03.002

[10] Rowart P, Erpicum P, Detry O, et al. Mesenchymal stromal cell therapy in ischemia/reperfusion injury. J Immunol Res. 2015;2015:602597. 26258151 10.1155/2015/602597 PMC4518154

[11] Haga H, Yan IK, Takahashi K, et al. Extracellular vesicles from bone marrow-derived mesenchymal stem cells improve survival from lethal hepatic failure in mice. Stem Cells Transl Med. 2017 Apr;6(4):1262–1272. 28213967 10.1002/sctm.16-0226 PMC5442843

[12] Lotfinia M, Kadivar M, Piryaei A, et al. Effect of secreted molecules of human embryonic stem cell-derived mesenchymal stem cells on acute hepatic failure model. Stem Cells Dev. 2016 Dec 15;25(24):1898–1908. 27676103 10.1089/scd.2016.0244 PMC5165664

[13] Chen YX, Zeng ZC, Sun J, et al. Mesenchymal stem cell-conditioned medium prevents radiation-induced liver injury by inhibiting inflammation and protecting sinusoidal endothelial cells. J Radiat Res. 2015 Jul;56(4):700–708. 26070321 10.1093/jrr/rrv026 PMC4497399

[14] Zagoura DS, Roubelakis MG, Bitsika V, et al. Therapeutic potential of a distinct population of human amniotic fluid mesenchymal stem cells and their secreted molecules in mice with acute hepatic failure. Gut. 2012 Jun;61(6):894–906. 21997562 10.1136/gutjnl-2011-300908

[15] Van Poll D, Parekkadan B, Cho CH, et al. Mesenchymal stem cell-derived molecules directly modulate hepatocellular death and regeneration in vitro and in vivo. Hepatology. 2008 May;47(5):1634–1643. 18395843 10.1002/hep.22236

[16] Xagorari A, Siotou E, Yiangou M, et al. Protective effect of mesenchymal stem cell-conditioned medium on hepatic cell apoptosis after acute liver injury. Int J Clin Exp Pathol. 2013;6(5):831–840. 23638214 PMC3638093

[17] Herrera MB, Fonsato V, Bruno S, et al. Human liver stem cells improve liver injury in a model of fulminant liver failure. Hepatology. 2013 Jan;57(1):311–319. 22829291 10.1002/hep.25986

[18] Chen J, Qiu M, Dou C, et al. MicroRNAs in bone balance and osteoporosis. Drug Dev Res. 2015 Aug;76(5):235–245. 26218893 10.1002/ddr.21260

[19] Zhong X, Coukos G, Zhang L. miRNAs in human cancer. Methods Mol Biol. 2012;822:295–306. 22144208 10.1007/978-1-61779-427-8_21 PMC4076826

[20] Clark EA, Kalomoiris S, Nolta JA, et al. Concise review: microRNA function in multipotent mesenchymal stromal cells. Stem Cells. 2014 May;32(5):1074–1082. 24860868 10.1002/stem.1623 PMC10668871

[21] Ebert MS, Sharp PA. Roles for microRNAs in conferring robustness to biological processes. Cell. 2012 Apr 27;149(3):515–524. 22541426 10.1016/j.cell.2012.04.005 PMC3351105

[22] Huang XP, Hou J, Shen XY, et al. MicroRNA-486-5p, which is downregulated in hepatocellular carcinoma, suppresses tumor growth by targeting PIK3R1. Febs J. 2015 Feb;282(3):579–594. 25475121 10.1111/febs.13167

[23] He J, Xiao B, Li X, et al. MiR-486-5p suppresses proliferation and migration of hepatocellular carcinoma cells through downregulation of the E3 ubiquitin ligase CBL. Biomed Res Int. 2019;2019:2732057. 10.1155/2019/2732057 PMC6949685 31976317

[24] Luo Q, Zhu J, Zhang Q, et al. MicroRNA-486-5p promotes acute lung injury via inducing inflammation and apoptosis by targeting OTUD7B. 2020 Jan 15. 10.1007/s10753-020-01183-3 31940107

[25] Hardiany NS, Yo EC, Ngadiono E, et al. Gene expression of molecules regulating apoptotic pathways in glioblastoma multiforme treated with umbilical cord stem cell conditioned medium. Malays J Med Sci. 2019 Nov;26(6):35–45. 31908585 10.21315/mjms2019.26.6.4 PMC6939736

[26] Iwatani S, Yoshida M, Yamana K, et al. Isolation and characterization of human umbilical cord-derived mesenchymal stem cells from preterm and term infants. J Vis Exp. 2019 Jan;26(143):e58806. 10.3791/58806 30741254

[27] Shojaeian A, Mehri-Ghahfarrokhi A, Banitalebi-Dehkordi M. Migration gene expression of human umbilical cord mesenchymal stem cells: a comparison between monophosphoryl lipid A and supernatant of lactobacillus acidophilus. Int J Mol Cell Med. 2019;8(2): 154–160. Spring. 32215266 10.22088/IJMCM.BUMS.8.2.154 PMC7081079

[28] Li J, Chen Y, Qin X, et al. MiR-138 downregulates miRNA processing in HeLa cells by targeting RMND5A and decreasing Exportin-5 stability. Nucleic Acids Res. 2014 Jan;42(1):458–474. 24057215 10.1093/nar/gkt839 PMC3874158

[29] Sato C, Yamamoto Y, Funayama E, et al. Conditioned medium obtained from amnion-derived mesenchymal stem cell culture prevents activation of keloid fibroblasts. Plast Reconstr Surg. 2018 Feb;141(2):390–398. 29369991 10.1097/PRS.0000000000004068

[30] Azizi R, Salemi Z, Fallahian F, et al. Inhibition of didscoidin domain receptor 1 reduces epithelial-mesenchymal transition and induce cell-cycle arrest and apoptosis in prostate cancer cell lines. J Cell Physiol. 2019 Nov;234(11):19539–19552. 30963567 10.1002/jcp.28552

[31] Livak KJ, Schmittgen TD. Analysis of relative gene expression data using real-time quantitative PCR and the 2(-delta delta C(T)) method. Methods. 2001 Dec;25(4):402–408. 11846609 10.1006/meth.2001.1262

[32] Tam SY, Wu VWC, Law HKW, et al. Low oxygen level induced epithelial-mesenchymal transition and stemness maintenance in colorectal cancer cells. Cancers (Basel). 2020 Jan 16;12(1):224. 10.3390/cancers12010224 PMC7017419 31963305

[33] Ebeid DE, Khalafalla FG, Broughton KM, et al. Pim1 maintains telomere length in mouse cardiomyocytes by inhibiting TGFβ signaling. Cardiovasc Res. 2021 Jan 1;117(1):201-211. 10.1093/cvr/cvaa066 PMC7797214 32176281

[34] Gottlieb RA, Pourpirali S. Lost in translation: miRNAs and mRNAs in ischemic preconditioning and ischemia/reperfusion injury. J Mol Cell Cardiol. 2016 Jun;95:70–77. 26582464 10.1016/j.yjmcc.2015.11.011 PMC4865448

[35] Nallamshetty S, Chan SY, Loscalzo J. Hypoxia: a master regulator of microRNA biogenesis and activity. Free Radic Biol Med. 2013 Sep;64:20–30. 23712003 10.1016/j.freeradbiomed.2013.05.022 PMC3762925

[36] Wen Z, Zheng S, Zhou C, et al. Bone marrow mesenchymal stem cells for post-myocardial infarction cardiac repair: microRNAs as novel regulators. J Cell Mol Med. 2012 Apr;16(4):657–671. 22004043 10.1111/j.1582-4934.2011.01471.x PMC3822837

[37] Ophelders DR, Wolfs TG, Jellema RK, et al. Mesenchymal stromal cell-derived extracellular vesicles protect the fetal brain after hypoxia-ischemia. Stem Cells Transl Med. 2016 Jun;5(6):754–763. 27160705 10.5966/sctm.2015-0197 PMC4878333

[38] Cantaluppi V, Gatti S, Medica D, et al. Microvesicles derived from endothelial progenitor cells protect the kidney from ischemia-reperfusion injury by microRNA-dependent reprogramming of resident renal cells. Kidney Int. 2012 Aug;82(4):412–427. 22495296 10.1038/ki.2012.105

[39] Sun XH, Wang X, Zhang Y, et al. Exosomes of bone-marrow stromal cells inhibit cardiomyocyte apoptosis under ischemic and hypoxic conditions via miR-486-5p targeting the PTEN/PI3K/AKT signaling pathway. Thromb Res. 2019;177:23–32. 30844685 10.1016/j.thromres.2019.02.002

[40] Cao L, Wang F, Li S, et al. PIM1 kinase promotes cell proliferation, metastasis and tumor growth of lung adenocarcinoma by potentiating the c-MET signaling pathway. Cancer Lett. 2019 Mar 1;444:116–126. 30583073 10.1016/j.canlet.2018.12.015

[41] Gao X, Liu X, Lu Y, et al. PIM1 is responsible for IL-6-induced breast cancer cell EMT and stemness via c-myc activation. Breast Cancer. 2019 Sep;26(5):663–671. 30989585 10.1007/s12282-019-00966-3 PMC6694096

[42] Zhu HH, Wang XT, Sun YH, et al. Pim1 overexpression prevents apoptosis in cardiomyocytes after exposure to hypoxia and oxidative stress via upregulating cell autophagy. Cell Physiol Biochem. 2018;49(6):2138–2150. 30257237 10.1159/000493817

[43] Meng XM, Tang PM, Li J, et al. TGF-β/Smad signaling in renal fibrosis. Front Physiol. 2015;6:82. 25852569 10.3389/fphys.2015.00082 PMC4365692

[44] Abudukeyoumu A, Li MQ, Xie F. Transforming growth factor-β1 in intrauterine adhesion. Am J Reprod Immunol. 2020 May;7:e13262. 10.1111/aji.13262 32379911

[45] Wang XM, Liu XM, Wang Y, et al. Activating transcription factor 3 (ATF3) regulates cell growth, apoptosis, invasion and collagen synthesis in keloid fibroblast through transforming growth factor beta (TGF-beta)/SMAD signaling pathway. Bioengineered. 2021;Dec;12(1):117–126. 10.1080/21655979.2020.1860491 PMC8806324 33315500

[46] Li T, Zhao N, Lu J, et al. Epigallocatechin gallate (EGCG) suppresses epithelial-mesenchymal transition (EMT) and invasion in anaplastic thyroid carcinoma cells through blocking of TGF-β1/Smad signaling pathways. Bioengineered. 2019 Dec;10(1):282–291. 31311401 10.1080/21655979.2019.1632669 PMC6650192

[47] Ganai AA, Husain M. Genistein attenuates D-GalN induced liver fibrosis/chronic liver damage in rats by blocking the TGF-β/Smad signaling pathways. Chem Biol Interact. 2017 Jan;5(261):80–85. 10.1016/j.cbi.2016.11.022 27876602

[48] Wang DT, Huang RH, Cheng X, et al. Tanshinone IIA attenuates renal fibrosis and inflammation via altering expression of TGF-β/Smad and NF-κB signaling pathway in 5/6 nephrectomized rats. Int Immunopharmacol. 2015 May;26(1):4–12. 25744602 10.1016/j.intimp.2015.02.027

[49] Ge Y, Cheng R, Sun S, et al. Fangxiao formula alleviates airway inflammation and remodeling in rats with asthma via suppression of transforming growth factor-β/Smad3 signaling pathway. Biomed Pharmacother. 2019;119:109429. 31505422 10.1016/j.biopha.2019.109429

